# Assessing Hydropower Potential in Nepal's Sunkoshi River Basin: An Integrated GIS and SWAT Hydrological Modeling Approach

**DOI:** 10.1155/2024/1007081

**Published:** 2024-01-23

**Authors:** Rinki Bhattarai, Binaya Kumar Mishra, Deepa Bhattarai, Dipendra Khatiwada, Pankaj Kumar, Gowhar Meraj

**Affiliations:** ^1^School of Engineering, Pokhara University, Pokhara, Nepal; ^2^Himalayan Institute of Science and Technology, Purbanchal University, Kathmandu, Nepal; ^3^Institute for Global Environmental Strategies, Hayama 240-0115, Kanagawa, Japan; ^4^Department of Ecosystem Studies, Graduate School of Agricultural and Life Sciences, The University of Tokyo, 1-1-1 Yayoi, Tokyo 113-8654, Japan

## Abstract

This study assessed the hydropower potential of a mountain watershed within the Sunkoshi River basin in Sindhupalchok, Nepal, utilizing geographic information systems (GIS) and the soil and water assessment tool (SWAT) hydrological model. Topographical, soil, land use, meteorological, and discharge data were employed to assess the study area for the appropriateness of hydropower generation. SWAT was utilized to delineate the Sunkoshi basin into 23 distinct subbasins and involved the creation of a detailed river network, incorporating various hydrological attributes including stream links, stream order, stream length, and slope gradient. After that, it was employed to simulate river discharges within these subbasins. The Sequential Uncertainty Fitting Version 2 (SUFI-2) algorithm, integrated within the SWAT Calibration and Uncertainty Program (SWAT-CUP), was employed to calibrate and validate the model. This step involved the adjustment of 25 selected parameters to enhance the model's accuracy and reliability in representing the hydrological processes of the Sunkoshi basin. Model performance was assessed utilizing three well-established efficiency criteria: coefficient of determination (*R*^2^ = 0.79), Nash–Sutcliffe efficiency (NSE = 0.73), and percent bias (PBIAS = 17.59). The study identified 36 sites across streams of order 3, 4, and 5 as having potential for hydropower generation. The hydropower potential at each identified site was evaluated using estimated stream flow and topographical head at various probability of exceedance (PoE) levels (40%, 45%, 50%, and 60%). The aggregate hydropower potential of the basin was quantified, yielding a potential of 371.30 MW at a 40% PoE. The findings suggest that an integrated approach combining SWAT-based hydrological modeling within a GIS can accurately assess a river basin's hydropower potential and provide insights into further evaluation of the comprehensive environmental assessment of the fragile Himalayan watersheds.

## 1. Introduction

With the global population rising and urbanization advancing rapidly, there is an increasing pressure on natural resources, fueling a heightened demand for sustainable energy sources throughout the globe [1]. This scenario is fostering the expansion of renewable energy sectors, particularly hydropower projects in the mountainous basins such as within the Himalayas, and is gaining particular attention for their potential to contribute to this growing energy demand [2]. Among various renewable options, hydropower is recognized as a well-established source, known for its clean and reliable energy production. This aligns well with both national and international policies aimed at environmental conservation and energy sustainability [3]. Hydropower, in essence, capitalizes on the kinetic energy of flowing water, predominantly from rivers and streams, driven by natural gravitational forces [4].

Nepal, a country in the central Himalayan range, characterized by unique topographical features and perennial river systems, holds substantial promise for hydropower generation [5]. The country's diverse topography, with elevations ranging between 60 and 8,849 msl in a narrow north-south extension of 145 and 248 km, provides steep topographic gradients conducive to hydropower generation [6]. With an estimated 83,500 MW hydropower potential, and receiving an average annual precipitation of 1,790 mm [7], it is home to numerous rivers and rivulets that originate from both the high Himalayas and the Mahabharat hill range [8]. To capitalize on its vast hydropower potential, comprehensive methodologies are being actively developed, taking into consideration the technologies such as remote sensing, information technology, and geographic information systems (GIS) [9]. These technologies when integrated with hydrological models offer multifaceted applications suited for hydropower generation evaluation such as catchment areas/drainage patterns delineation and the estimation of hydropower potential [10]. Using these technologies, and with the availability of updated land use, topographical information, and other relevant administrative datasets [11], hydropower capacity can now be calculated more efficiently, aimed at understanding the economic and sustainability aspects, without the need for site visits [12].

In the realm of hydropower potential evaluation science, it is essential to differentiate between gross, technical, and economic hydropower potentials. Gross hydropower potential refers to the theoretical maximum amount of energy that could be harnessed from a particular river [13]. Technical potential is the portion of the gross potential that can be generated on the basis of existing site conditions, legal constraints, technological capabilities, and infrastructure [14]. The last one, economic potential is a subset of the technical potential that is both financially and economically viable for development [15]. The primary aim of this research is to evaluate the potential for hydropower generation in the Sunkoshi River basin, Nepal, through an integrated approach using RS, GIS, and hydrological modeling. The aim is to assess all the three hydropower potentials. To achieve this, we performed an in-depth analysis of the study area's hydrological and topographical characteristics to ascertain its suitability for hydropower generation. We employed RS and GIS in association with the SWAT model, specifically focusing on assessing the gross, technical, and economic potentials of hydropower in the region. In addition to providing the planners and decision-makers an overall picture of the energy potential of the region, this finding of this work shall also serve as a baseline analysis for future research on the potential environmental impacts of hydropower development.

## 2. Study Area

**Figure 1:**
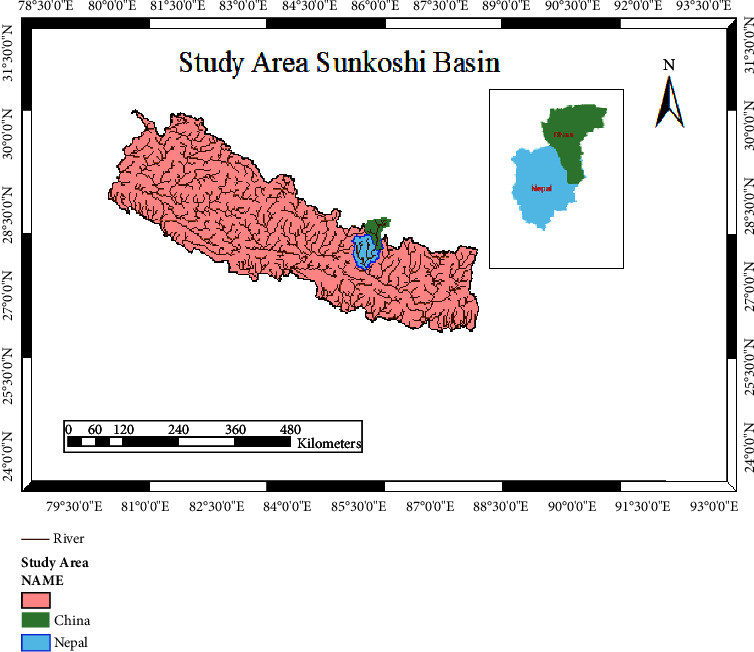
The location of the study area with respect to Nepal.

This research focuses on the Sunkoshi River basin, situated in Sindhupalchok, Nepal. The basin is a transboundary entity extending through both Tibet (China) and Nepal. In Tibet, the upper Sunkoshi River section is known as pique. The Bhotekoshi, originating in Tibet, joins the Sunkoshi River after traversing some miles south of the Arun River watershed. This basin is part of the Sapta Koshi River system in Nepal. Additionally, the Indrawati River, originating from the Gosaikunda's eastern watershed and commencing its flow from Langtang National Park, merges with Melamchi Khola at Dolalghat. Geographically, the Sunkoshi River catchment is delineated by longitudes ranging from 86°19′28.41″ E to 85°26′40.76″ E and latitudes spanning from 28°29′51.54″ N to 27°31′34.39″ N. Elevations within the basin vary from 588 m to 7945 m above sea level, as indicated in Figure 1. Climatically, temperatures fluctuate between a high of 33°C in summer and dip below 4°C in winter. The basin's total area is about 4812 km^2^, divided between Tibet (2041 km^2^) and Nepal (2771 km^2^). The basin's morphology is leaf-shaped, with 110 km mean areal length and 66 km width.

## 3. Materials and Methods

### 3.1. Data

This research utilized two primary categories of data: spatial data and time series. The first type comprised of three key components: (i) digital elevation model (DEM) to characterize the region's terrain, (ii) land use/land cover (LULC) to assess the surface characteristics, and (iii) soil data to analyze soil types and compositions. In the second type, we employed (i) weather data, which included parameters such as precipitation, solar radiation, temperature, wind velocity, and relative humidity, and (ii) daily discharge data, which was important for the calibration cum validation of the hydrological model. The sources of all these datasets are detailed in Table 1. Before their application in the study, all data underwent a thorough preprocessing and reformatting procedure to ensure compatibility and accuracy for use as input in the subsequent analyses.

#### 3.1.1. DEM

**Figure 2:**
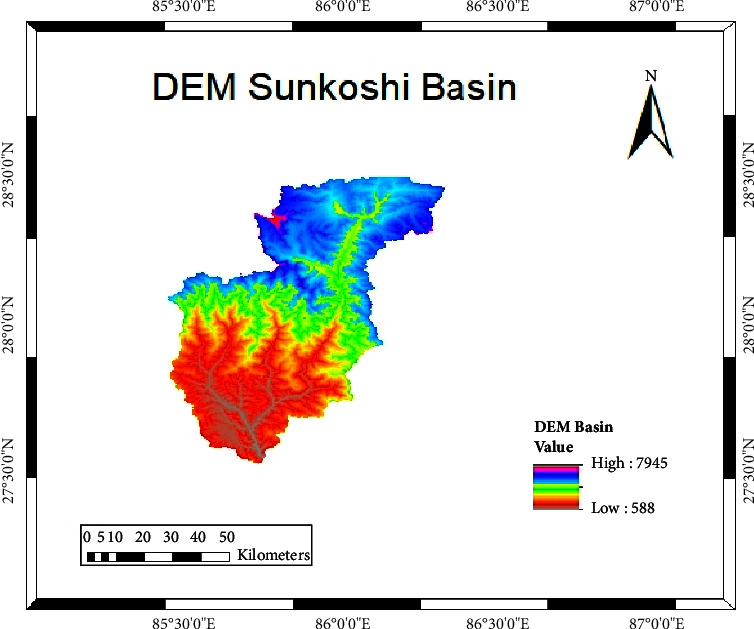
Elevation profile of the Sunkoshi basin.

We used Shuttle Radar Topography Mission (SRTM) DEM of 30 m resolution. The elevation of the study area is between 588 m and 7945 m asl (Figure 2).

#### 3.1.2. LULC

**Figure 3:**
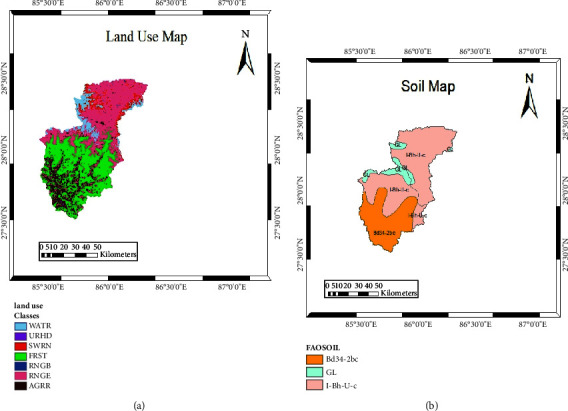
(a) Land use of Sunkoshi basin and (b) soil classification of Sunkoshi basin.

The LULC raster map for the study was obtained from the Land Cover Climate Change Initiative (CCI), Climate Research Data Package (CRDP) of 2015, focusing on the Asian region. The relevant segment of this map was extracted using the ArcGIS Spatial Analyst tool, guided by the boundary polygon of the Sunkoshi basin watershed. Within the study area, six distinct land-use classes were characterized. Specifically, water bodies (WATR) constitute 7.137% of the basin area, high-density urban areas (URHD) make up 0.079%, the southwestern range (SWRN) comprises 4.615%, forested areas (FRST) account for 33.304%, range scrubland (RNGB) represents 0.145%, grassland or herbaceous areas (RNGE) cover 39.333%, and agricultural land designated for row crops (AGRR) occupies 12.384% of the basin. The distribution of these land-use classes is visually represented in Figure 3(a).

#### 3.1.3. Soil

Three distinct soil types were mapped within the study area. The most prevalent is the l-Bh-U-C soil unit, which encompasses 62.66% of the basin area. In contrast, the Bd34-2bc soil unit is found predominantly in the lower part of the basin and covers 31.41% of that area. This soil unit has various properties, and these properties are associated with certain soil parameters such as SOL_AWC (available water capacity of the soil layer), SOL_AWD (moist soil albedo), SOL_K (saturated hydraulic conductivity), SOL_Z (depth from the soil surface to bottom of the layer), and SOL_BD (moist bulk density). All these parameters are used for a process called “model calibration.” Study areas' detailed soil maps are shown in Figure 3(b).

#### 3.1.4. Precipitation

**Figure 4:**
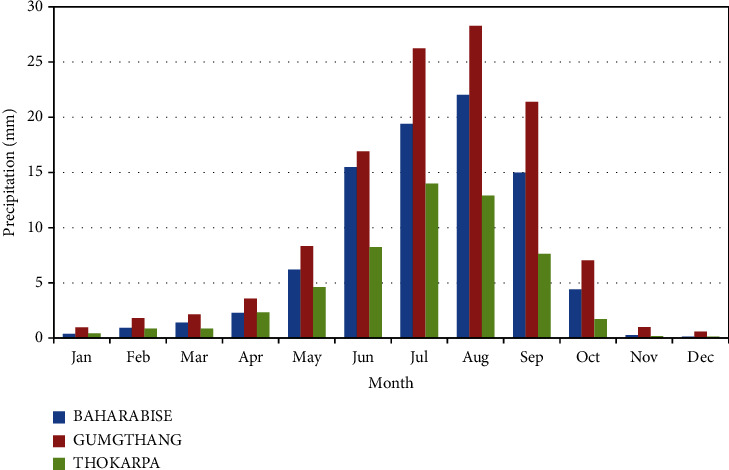
Observed monthly rainfall at three stations.

For the Sunkoshi basin, daily precipitation data were obtained from the three stations of rain gauges as shown in Table 1. The data cover the period from 2000 to 2017 and are illustrated in Figure 4.

#### 3.1.5. Temperature

**Figure 5:**
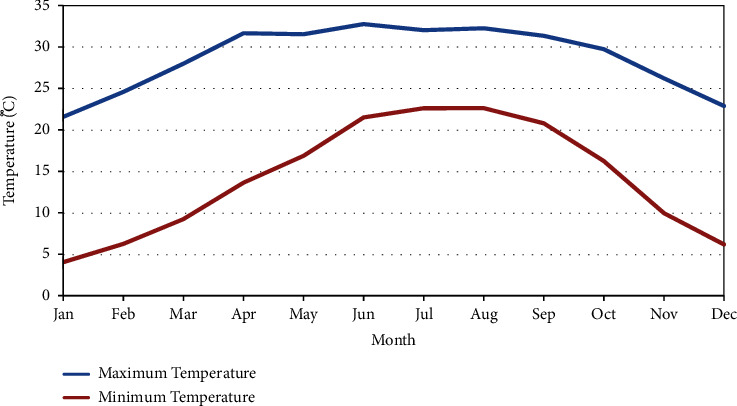
Mean monthly minimum and maximum temperatures at Panchkhal station.

Daily observed temperature data were retrieved from Panchkhal station, located within the basin, for the period spanning 2000 to 2017. The recorded *T*_min_ and *T*_max_ were −2.1°C and 39°C, respectively. The mean monthly minimum and maximum temperatures observed at Panchkhal station are illustrated in Figure 5.

#### 3.1.6. Discharge

**Figure 6:**
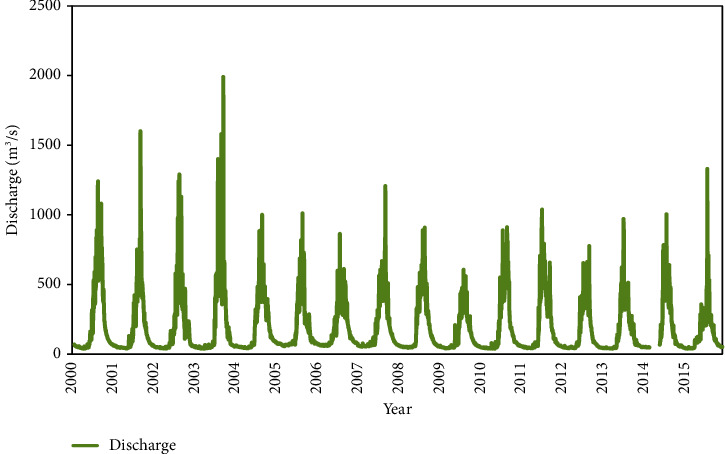
Discharge data at Pachuwarghat station.

Data for daily discharge for the Sunkoshi River were collected at the Pachuwarghat station (station no. 630) for the period from 2000 to 2015, as depicted in Figure 6.

### 3.2. Methodology

**Figure 7:**
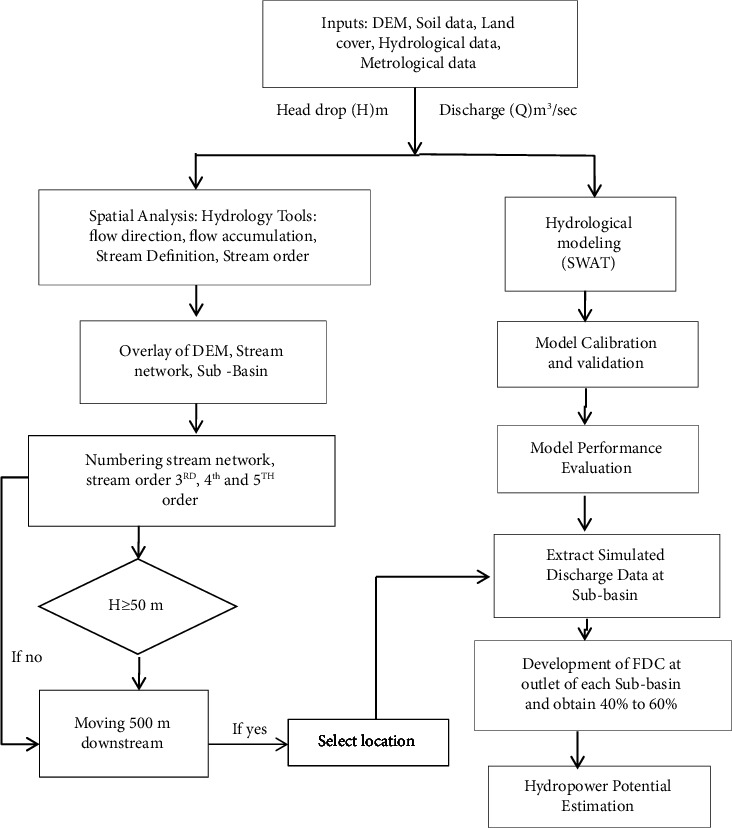
Methodology for assessment of hydropower potential.

The essential factors for estimating hydropower potential are topographic head and river flow [16].The methodology for these estimations is broadly categorized into two main components, discharge evaluation within the river system and potential head drop identification. This method has gained significant traction in recent assessments of hydropower potential.  Figure 7 presents the flowchart employed in this research to evaluate the river's hydropower potential.

#### 3.2.1. Identification Criteria for Sites

For the purpose of potential hydropower site identification, a specific set of criteria was established:

- Stream Order: Only streams of 5th, 4th, and 3rd orders are considered to ensure adequate water flow [17]. Higher-order streams provide more steady and dependable water flow all year. As a result, they are more suitable for continuous power generation, which is required for a stable energy supply. The environmental impact of hydropower projects may be reduced by focusing on higher-order streams. Smaller streams are often more ecologically sensitive, and developing hydropower on these smaller streams can have a more significant impact on local ecosystems.
- Head Availability: A minimum head of 50 meters can be adjusted to ensure that there is enough potential energy to generate a respectable quantity of power.
- Minimum Interval between Sites: It is a regulatory requirement to maintain a minimum distance of 500 meters between two adjacent hydropower facilities [7]. This condition is crucial for ensuring a physical separation between the tailrace area of one facility (where water exits the hydropower plant) and the diversion structure of the next facility (where water is redirected into the plant for power generation). This mandated separation distance is vital for the ecological health of the river. It allows a stretch of the river between the two facilities to recover and rejuvenate, supporting the maintenance and restoration of the river's natural ecosystem. When hydropower operations disrupt the natural flow of a river, creating this gap helps to minimize the negative ecological impacts by providing the ecosystem with time and space to recover and return to a state of equilibrium. In essence, it is a measure to balance the environmental effects of hydropower generation with the preservation of the river's health and biodiversity.
- Environmental Constraints: Areas under the classification of national parks or wildlife reserves are excluded from consideration.
- Ongoing Development Projects: Existing development projects within the study area are also considered for site selection.

**Figure 8:**
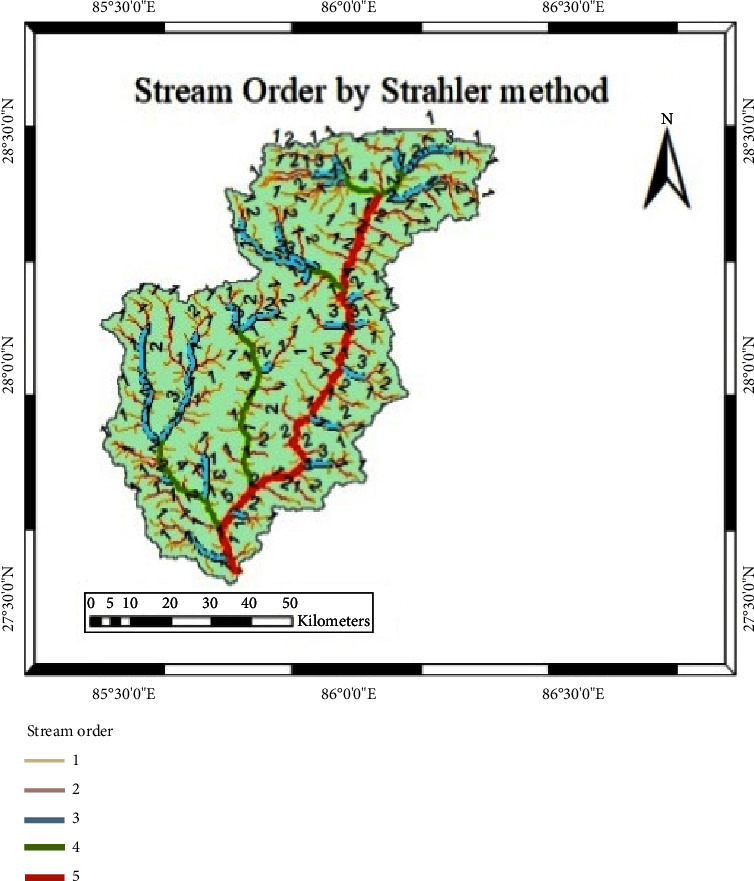
Stream order by Strahler method.

To classify the streams, the Strahler method was employed, as illustrated in Figure 8. According to this approach, a first-order stream originates at the highest elevation, while the confluence of two first-order streams results in a second-order stream; this classification continues down to the last stream in the watershed [11, 18]. The existing stream network and DEM were overlaid on each other to investigate the elevation and the available drop along the stream bed. Site evaluations commenced from the terminus of the highest-order stream and continued downstream until reaching the watershed's final outlet. Decisions regarding suitable hydropower sites were made in accordance with the established criteria [19].

#### 3.2.2. Discharge Analysis

For the river discharge simulation, we utilized SWAT. The latter is designed to operate on diverse watershed scales and is capable of simulating the environmental consequences of various land uses, land management strategies, and climatic changes. It accomplishes this by estimating both the quality and quantity of groundwater and surface [20]. Recognized for its flexibility and robustness, the SWAT model is well-suited for simulating a diverse array of watershed scenarios. The model accounts for a myriad of hydrological factors including sediment transport, surface runoff, percolation, reservoir storage, and groundwater flow. It discretizes the watershed into interconnected subbasins, which are segmented into hydrological response units (HRUs). These HRUs are characterized by uniform soil types, land usage, and slopes, and they exhibit comparable hydrological behavior. SWAT modeling consists of two primary phases: the land phase and the routing phase [21]. The routing phase governs the transit of water, sediment, and nutrients to the watershed outlet, while the land phase controls the sediment, pesticides, quality of the flow, and nutrients that enter the main channel. A water balance equation is used by the land phase to account for various hydrological constituents such as precipitation, runoff, percolation, evapotranspiration, and return flow [22].

(1) $$\begin{array}{c} {\text{SW}}_{T}={\text{SW}}_{O}+\overset{t}{\underset{i=1}{\sum }}\left(R_{\text{day}}-Q_{\text{surf}}-E_{a}-W_{\text{seep}}-Q_{\text{gw}}\right), \end{array}$$

where SW_*t*_ is the final soil water content (mm H_2_O), SW_*o*_ is initial soil water content on day *i* (mm H_2_O), *R*_day_ is the precipitation amount on day *i* (mm H_2_O), *Q*_surf_ is the surface runoff amount on day *i* (mm H_2_O), *E*_*a*_ is the evapotranspiration amount on day *i* (mm H_2_O), *W*_seep_ is the water amount entering the vadose zone from the soil profile on day *i* (mm H_2_O), and *Q*_gw_ is the return flow amount on day *i* (mm H_2_O). The SWAT model was chosen based on the accessibility of the model's data requirements user-friendliness, the cost of the tools, and user support elements. Arc SWAT 10.3 version was utilized to build the hydrological model. ArcSWAT is a version of SWAT that runs on ArcMAP.

#### 3.2.3. Calibration and Validation

The SWAT-CUP interface, specifically designed for the SWAT model, facilitates this process [23]. This user-friendly interface enables seamless integration with any calibration, uncertainty, or sensitivity program designed for SWAT [24]. For the purposes of this study, the most recent version of SWAT-CUP, namely, version 5.1.6, was utilized to perform both calibration and validation exercises. The SWAT-CUP links SUFI-2, ParaSol, GLUE, PSO, and MCMC algorithms to SWAT [25]. Among the above algorithms, SUFI-2 is more frequently used for performing sensitivity analysis, parameterization, calibration/validation, and uncertainty analysis of the hydrological parameters [26]. This owes to the fact that this algorithm has less tedious calibration processing features to perform within realizable time bounds, as well as the parameter availability for modeling water balance. It also accounts well for uncertainties, and the less number of iterations required for achieving better prediction uncertainty bands to aid in the best model performance [27, 28]. Calibration, validation, and sensitivity analyses were executed within the framework of the SWAT-CUP, employing the SUFI-2 algorithm. The standard hydrological simulations of SWAT are based predominantly on terrestrial factors such as precipitation, land cover, and soil types. In glacier-influenced watersheds, however, it is critical to integrate the distinct processes of glacier melting. To this end, we have incorporated a modified energy balance approach within SWAT to simulate glacier melt. This approach considers key factors influencing glacier melt rates, such as solar radiation, relative humidity, wind speed, and air temperature. The model calculates melt rates by assessing the energy absorbed and released by the glacier surface, providing a more dynamic and responsive representation of glacier hydrology. This was performed by fine-tuning SWAT parameters to reflect the unique hydrological characteristics of glacier-fed streams. These parameters included groundwater recession coefficient (ALPHA_BF), surface runoff lag time (SURLAG), and soil environmental factor (ESCO), which are pivotal in simulating the delayed and extended runoff typically associated with glacier melt. Moreover, we also established temperature thresholds specific to snow and glacier melting processes, ensuring that the model accurately represents the seasonal and temporal variations in glacier-fed streamflows.

After constructing the model, calibration is conducted by fine-tuning the parameters of the model within their recommended boundaries to align the simulated outcomes with observed data. The calibrated model must be validated before its simulation performance may be tested. The calibrated model was validated using a separate set of meteorological and discharge data. The validation process was conducted in accordance with guidelines recommended in the existing literature [29]. Sensitivity analysis was performed to investigate the relationship between variable changes in model inputs and outputs. It provides an approach for investigating the model's response in a way that eliminates the influence of error due to the natural variation of the input parameters of the model. SUFI-2 helps to balance the objective function after every run to locate the best simulation [30].

#### 3.2.4. Performance Evaluation

The model's performance was evaluated by utilizing both graphical and statistical model evaluation techniques. In statistical evaluation techniques, PBIAS, NSE, and coefficient of determination (*R*^2^) were utilized. The graphical technique gives a visual comparison of simulated and observed constituent data as well as a preliminary review of model performance.

(2) $$\begin{array}{c} \text{NSE}=1-\left[\frac{\overset{n}{\underset{i=1}{\sum }}{\left(y_{i}^{\text{obs}}-Y_{i}^{\text{sim}}\right)}^{2}}{\overset{n}{\underset{i=1}{\sum }}{\left(y_{i}^{\text{obs}}-Y^{\text{mean}}\right)}^{2}}\right], \end{array}$$

where *y*_*i*_^obs^ is *i*th observations for the constituent being evaluated, *Y*_*i*_^sim^ is the *i*th simulated value for the constituent being evaluated, *Y*^mean^ is the mean of the observed data for the constituent being evaluated, and *n* is the total number of observations.

(3) $$\begin{array}{c} R^{2}={\left(\frac{\overset{n}{\underset{i=1}{\sum }}\left(O_{i\;}-\overset{¯}{O}\right)\left(P_{i\;}-\overset{¯}{P}\right)}{\sqrt{\overset{n}{\underset{i=1}{\sum }}{\left(O_{i\;}-\overset{¯}{O}\right)}^{2}}\sqrt{\overset{n}{\underset{i=1}{\sum }}{\left(P_{i\;}-\overset{¯}{P}\right)}^{2}}}\right)}^{2}, \end{array}$$

where *O* is observed and *P* is the simulated value.

(4) $$\begin{array}{c} \text{PBAIS}=\left[\frac{\overset{n}{\underset{i=1}{\sum }}\left(Y_{i}^{\text{obs}}-Y_{i}^{\text{sim}}\right)*100}{\overset{n}{\underset{i=1}{\sum }}\left(Y_{i}^{\text{obs}}\right)}\right], \end{array}$$

where *y*_*i*_^obs^ is *i*th observations for the constituent being evaluated and *Y*_*i*_^sim^ is the *i*th simulated value for the constituent being evaluated.

#### 3.2.5. Flow Duration Curve (FDC)

FDC is the cumulative frequency curve that depicts the percentage of time that the flow in the stream is most likely to reach some particular value of interest. Based on the flow retrieved from the SWAT, FDC was generated for all subbasins. After determining the discharge at Q40 of all subbasins of the study area, discharge at each intake of potential sites was calculated by the drainage area ratio (AR) method. This method presumes that the runoff per unit drainage area is equivalent through all hydrologically equivalent basins and is selected as the runoff transfer (RT) approach to simulate the catchment's runoff with ungauged discharge. Using cluster analysis techniques, watersheds with similar climate and physical characteristics were clustered together. As a result, the AR method could be used in those watersheds only that had similar climatic and physical features. The given day daily runoff in the AR approach is simulated as follows:

(5) $$\begin{array}{c} \frac{Q_{y,t}}{Q_{x,t}}=\frac{A_{y}}{A_{x}}, \end{array}$$

where *Q*_*y*,*t*_ is the simulated runoff of day-*t* of the target basin, *Q*_*x*,*t*_ is the observed runoff on day-*t* at the basin that donates water, *A*_*y*_ is the target catchment area, and *A*_*x*_ is the area of the basin that donates water [31].

#### 3.2.6. Hydropower Potential Estimation

The run-of-river (ROR) hydropower potential can be determined as and when the potential head drop and design discharge or flow exceedance have been calculated [32]. The total ROR of the basin is evaluated by summation of the ROR potential of all the sites.

(6) $$\begin{array}{c} P=\rho \;x\;g\;x\;Q\;x\;H, \end{array}$$

where *P* is the power produced in Watt (W), *ρ* is the mass density of water (kg/m^3^), *g* is acceleration due to gravity (m/s^2^), *Q* is the discharge (m^3^/s), and H is gross head drop (*m*). When there are *n* number of potential sites in a specified basin, the total power of each hydropower site is assessed by summation of the potential of all hydropower sites.

(7) $$\begin{array}{c} P=\overset{n}{\underset{i=1}{\sum }}\rho \;x\;g\;x\;Q\;x\;H, \end{array}$$

where *i* is the potential site number (*i* *=* 1…*n*), *n* is the number of potential sites, *ρ* is taken as 1000 kg/m^3^, and *g* is 9.81 m/s2. *H* is the elevation difference between the tailrace and headrace. By calculating *Q* and H of any potential site, the hydropower potential is estimated.

## 4. Results and Discussion

### 4.1. Potential Location of Hydropower Sites

**Figure 9:**
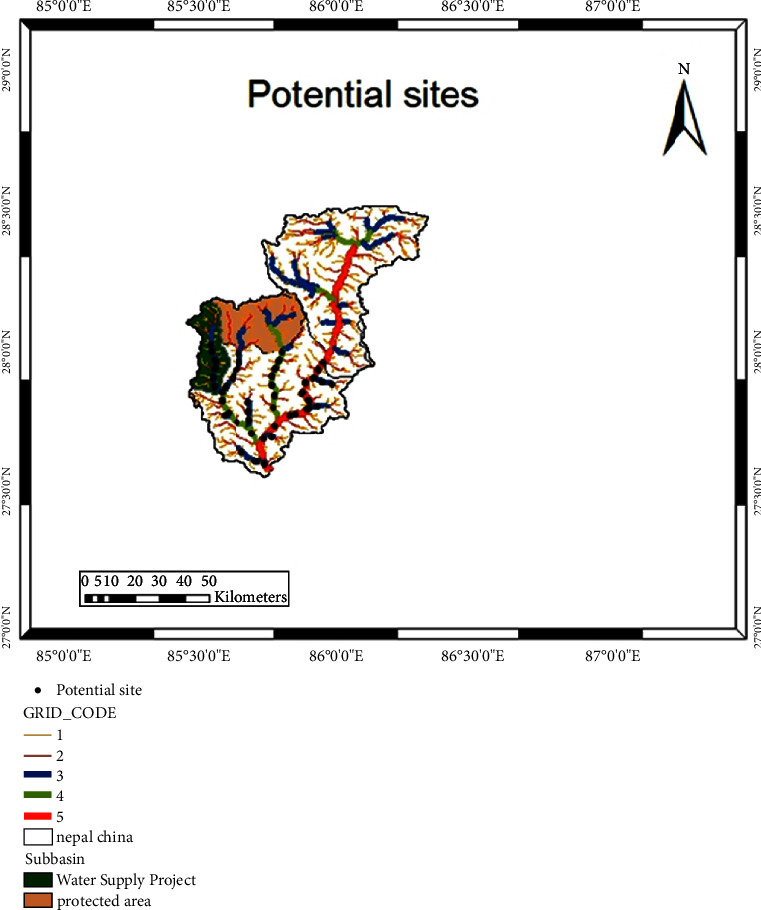
Location of potential sites for hydropower generation.

To identify potential hydropower sites, a multicriteria approach was adopted, incorporating the following considerations:

- *Order of Stream*: Only higher-order streams, specifically 5th-, 4th-, and 3rd-order streams, were considered to ensure an adequate flow of water.
- *Head Availability*: Each potential site was required to have a minimum head of 50 meters to be considered viable for hydropower generation.
- *Minimum Interval between Sites*: The distance between two consecutive hydropower sites was mandated to be no less than 500 meters to prevent overlapping impacts.
- *Environmental Constraints*: An area of 704 km^2^ in the upper left part of the basin is encompassed by Langtang National Park and was, therefore, excluded from consideration for hydropower development. This is illustrated in Figure 9.
- *Existing Development Projects*: The Melamchi River Water Diversion Project, which involves activities such as the construction of a water treatment plant (WTP) and water diversion tunnel (WDT) at Sundarijal, necessitated the deduction of 1.96 m^3^/s from the discharge in subbasin number 13.

Upon satisfying these criteria related to stream order, head availability, site spacing, environmental considerations, and existing development projects, the ArcGIS tool was employed to locate potential powerhouse and intake sites. In total, 36 schemes met the set criteria. The identified sites exhibited a range of gross head values between 50 and 591 meters. Notably, four out of the 33 potential sites possessed a gross head exceeding 250 meters.

### 4.2. Watershed Delineation

**Figure 10:**
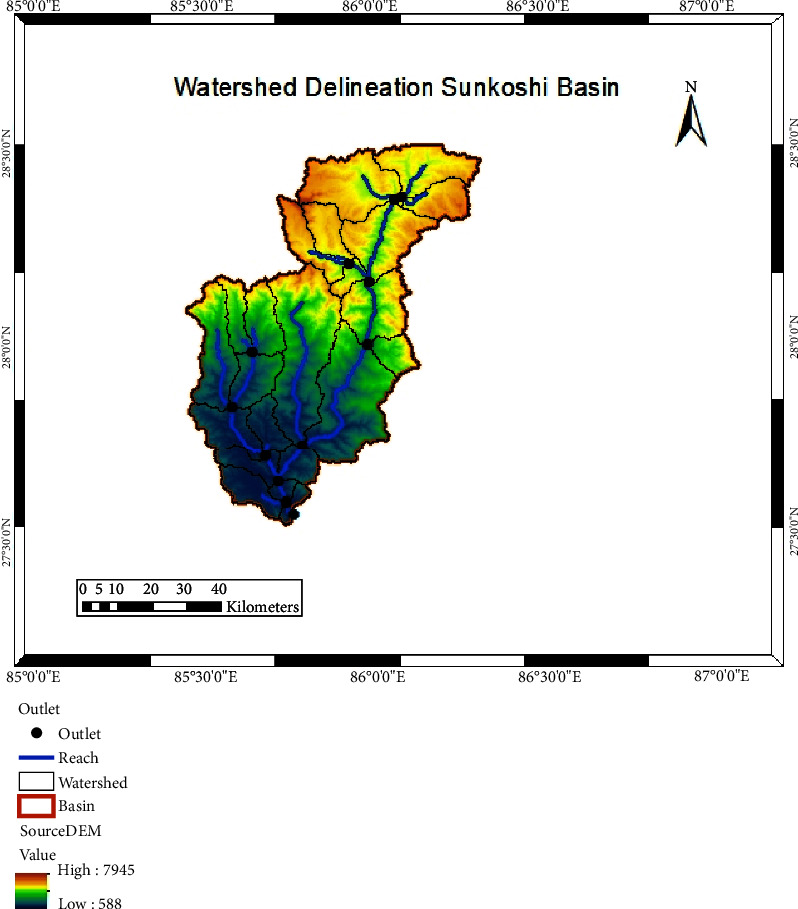
Watershed delineation of the study area.

During the process of watershed delineation, a specific threshold value of the stream network was utilized to characterize the stream network. Furthermore, due to the basin's steep gradient, five slope bands were introduced during the creation of hydrological response units (HRUs) [33]. The delineation resulted in a total of 286 HRUs and 23 subbasins within the Sunkoshi basin. According to the SWAT report, the basin covers a total catchment area of 4812.11 km^2^. Climatological data of the basin indicate that it receives a mean annual precipitation of 3133.8 mm. Hydrological parameters were also assessed: the surface runoff (*Q*) was calculated to be 937.64 mm, the lateral discharge amounted to 622.38 mm, and the groundwater discharge for both the shallow and deep aquifers was 655.75 mm and 32.79 mm, respectively. The spatial delineation of the watershed is illustrated in Figure 10.

### 4.3. Calibration, Validation, and Performance Evaluation

**Figure 11:**
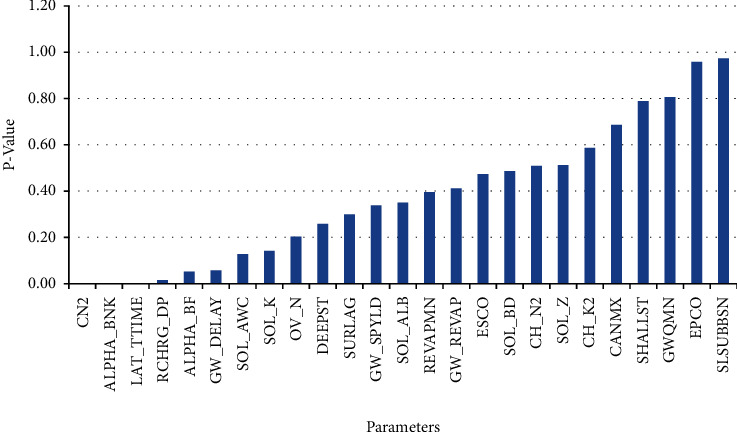
Sensitive parameters and their *p* value.

The hydrological model was calibrated utilizing daily flow data spanning the period from 2002 to 2009 and subsequently validated for the years from 2010 to 2015. The result of the sensitivity analysis of 25 parameters is provided in Table 2 (Figure 11). The output of the global sensitivity analysis with the *t*-test depicts the most sensitive parameters (having *p* < 0.05). In the calibration process using the NSE as the objective function, we identified four parameters as most sensitive: curve number II (CN2, *p* ≤ 0.001, *t* = 17.04), base flow alpha factor for bank storage (ALPHA_BNK, *p* ≤ 0.001, *t* = 10.79), lateral flow travel time (LAT_TIME, *p* ≤ 0.001, *t* = −3.29), and deep aquifer percolation fraction (RCHRG_DP, *p* < 0.02, *t* = −2.43). These parameters were found to be the most sensitive based on their significant *t*-values and low *p* values, indicating a strong statistical influence on the model's performance. To further refine the calibration, we conducted a sensitivity analysis over the period from 2002 to 2009. This analysis involved adjusting these four sensitive parameters within their plausible ranges using the SUFI-2 algorithm. For each parameter, 500 simulations were executed to ascertain their role in the predictive accuracy of the model. This extensive sensitivity analysis allowed us to understand how variations in these parameters over the seven-year period influenced the model's output, leading to their selection as the most sensitive for calibration purposes. The observed and predicted results were correlated at an equal time with FLOW_OUT_23 (subbasin 23), which was the output end.

**Figure 12:**
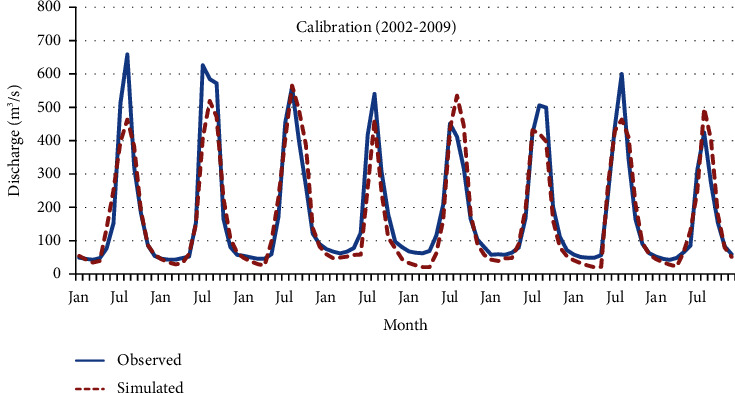
Observed and simulated monthly stream flow calibration hydrograph at Pachuwarghat station.

**Figure 13:**
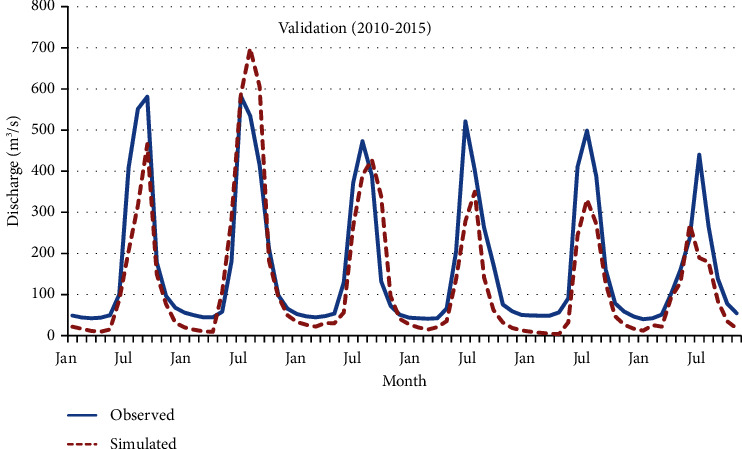
Observed and simulated monthly stream flow validation hydrograph at Pachuwarghat station.

Figures 12 and 13 graphically present the model's performance during both the calibration and validation phases at a single hydrological station.

Graphically, it can be observed that during calibration and validation, the model correctly simulated low flows but underestimated peak flows. The SWAT model does not accurately predict high-flow events, resulting in either underestimation or overestimation [34]. The model's predictive accuracy was evaluated using statistical indicators, aligning with the criteria suggested by earlier studies [8]. Specifically, the coefficient of determination (*R*^2^) exceeded 0.5, the NSE also surpassed 0.5, and the PBIAS was observed to be within ±25%. These metrics substantiate that the model's performance falls within acceptable ranges during both the calibration and validation phases at the designated hydrological station [35]. Detailed results of this statistical evaluation are catalogued in Table 3. The overall summary of the statistical evaluation of the model (Table 3) revealed that the model performed well.

### 4.4. Flow Duration Curve (FDC)

**Figure 14:**
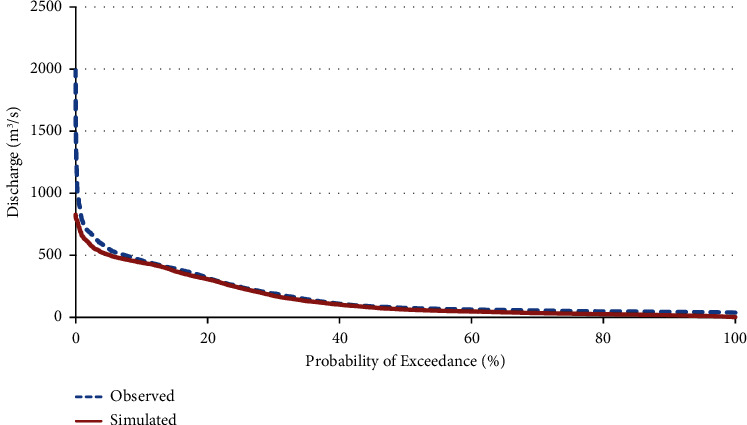
Flow duration curve of observed and simulated discharge at the outlet.

The Sunkoshi basin comprises 23 subbasins, approximately half of which are situated in Tibet, China. Of the total subbasins, 13 are located within Nepalese territory. For the purpose of this study, subbasin 11, subbasin 12 which are part of the Langtang National Park, were excluded from the hydropower potential assessment. Consequently, only 11 subbasins were selected for further analysis. The flow duration curve (FDC) for the basin outlet, featuring both simulated and observed discharges, is illustrated in Figure 14.

For run-of-river (RoR) hydropower projects, the Department of Electricity Development (DOED) typically considers a discharge value with 40% dependability for power estimation (DOED, 2018). This study expanded the scope to include dependability flows of 40%, 50%, 55%, and 60%. The estimated hydropower potential at varying levels of probability of exceedance (PoE) is tabulated accordingly. Hydropower potential at the identified locations was calculated based on standard power equations, incorporating parameters such as hydraulic head and FDC. Environmental flows—representing the water required to sustain the river ecosystem—were deducted from the calculated discharge [36]. This study adopted an environmental flow equivalent to 10% of the average monthly discharge, in alignment with Nepal's Hydropower Development Policy of 2001. Subsequently, the net discharge at each subbasin outlet, termed as Q40, was calculated at a plant operating efficiency of 90%. For instance, in subbasin 13, the Q40 discharge was reduced by 1.968 m^3^/s for the final power calculation.

### 4.5. Estimation of Hydropower Potential

**Figure 15:**
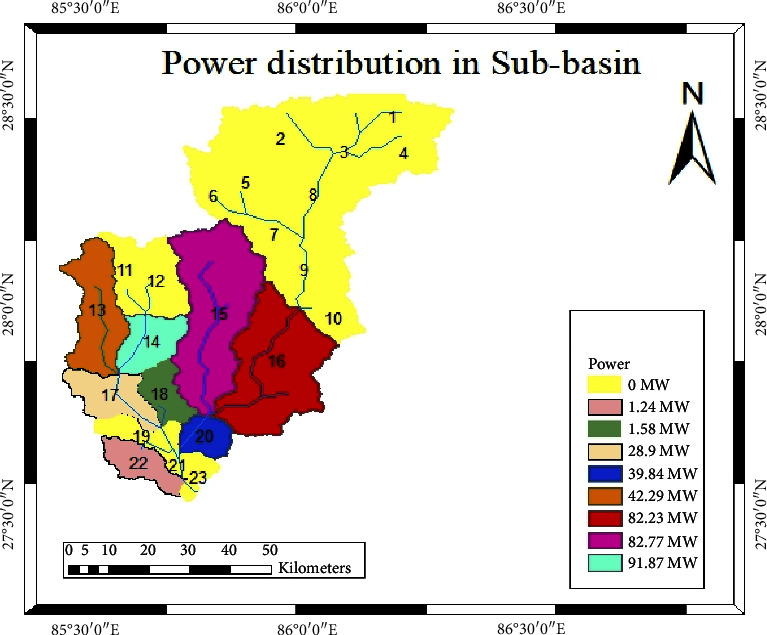
Hydropower potential distribution in subbasins.

The power output was initially calculated based on daily flow rates and subsequently estimated at a 40% probability of flow exceedance (Table 4). A comprehensive summary of the total power potential, along with its distribution across various subbasins, is provided in Table 4. According to the data presented, subbasin 14 exhibits the highest power potential, boasting a capacity of 91.87 MW. In contrast, subbasin 22 registers the lowest power potential, with a capacity of merely 1.20 MW. A graphical representation of the power distribution across subbasins can be found in Figure 15.

The potential for hydropower generation at a 40% probability of exceedance (PoE) was stratified into three categories based on the project's capacity within Nepal's Sunkoshi basin, as illustrated in Table 5. Out of the 36 identified sites, five were categorized as mini hydropower projects, contributing to 0.74% of the basin's total potential. Additionally, 16 sites were designated as small hydropower projects, accounting for 27.48% of the basin's cumulative potential. Lastly, 15 sites were classified as medium hydropower projects, making up 71.77% of the basin's overall potential for hydropower generation (Table 6) [37].

### 4.6. Comparison with DOED on Hydropower Assessments and Hydropower Sites

**Figure 16:**
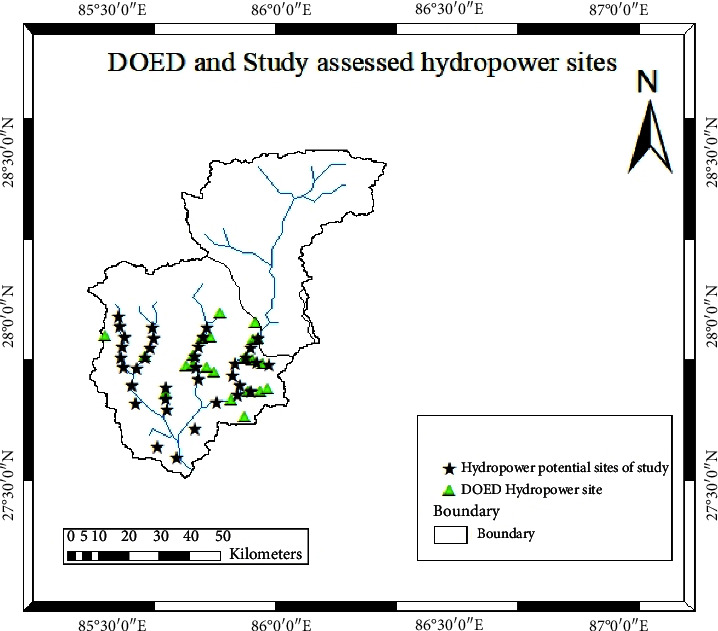
DOED and study assessed hydropower sites.

According to data from the Department of Electricity Development of Nepal (DOED), there are 39 sites identified for hydropower potential, encompassing various stages such as power plant installation, survey licensing, and construction licensing. Specifically, 11 hydropower sites with a combined capacity of 103.15 MW are currently operational, 13 sites with a total capacity of 148.9 MW have been issued survey licenses, and 17 sites with an overall capacity of 433.08 MW have received construction licenses. In contrast, the present study identifies 36 potential sites with a total estimated capacity of 371.30 MW within the Sunkoshi watershed (see Figure 16 for site distribution). Although the estimated power potential from this study is comparatively lower than the DOED's assessments, new sites with varying capacities have been identified. An examination of the geographical distribution indicates that some of the identified locations are in close proximity to DOED-assessed sites, while others are unique to this study. These newly identified sites were evaluated under different scenarios and offered additional insights that could serve as decision-making tools for selecting and identifying suitable run-of-river (RoR) hydropower schemes in the Sunkoshi basin [14, 38–40]

## 5. Conclusions

This study employed advanced hydrological models and GIS technologies to assess the run-of-river (RoR) hydropower potential within Nepal's Sunkoshi basin. The soil and water assessment tool (SWAT) and ArcGIS were chosen as the methodological frameworks for this evaluation. Utilizing available meteorological and discharge data, the study accomplished a detailed characterization of the watershed, enabling a comprehensive assessment of its hydropower potential. The model's robustness was confirmed through stringent evaluation criteria: a coefficient of determination (*R*^2^) of 0.79, NSE of 0.73, and a PBAIS of 17.59. Based on these metrics, 36 potential sites were identified, collectively offering a potential capacity of 371.30 MW at a 40% probability of flow exceedance. These findings are invaluable for hydropower developers, water resource planners, and policymakers for optimizing water resource utilization in the Sunkoshi basin. Moreover, the study offers insights that can guide future academic investigations into key factors influencing hydropower potential. Despite these promising results, the study was limited by the availability of hydrological stations and relevant meteorological data. Calibration of the entire watershed was constrained due to the singular hydrological station located downstream. The absence of meteorological data in higher elevations further complicated the calibration process. Thus, future efforts should focus on acquiring upstream data and expanding meteorological monitoring infrastructure. Such enhancements would facilitate more accurate hydrological modeling and could inform other meteorological and physiographic research within the basin. This study also underscores the need for additional research into the sustainability of hydropower developments, including impacts on local fisheries, sedimentation effects, and the potential cascade impacts resulting from hydropower projects.

## Figures and Tables

**Table 1:** Source of datasets.

Category	Data type	Data source
Climate	Precipitation and temperature	Department of Hydrological and Metrology (DHM), Nepal
Topography	DEM 30 m resolution	Shuttle Radar Topography Mission (SRTM)
Land use	LU map	https://maps.elie.ucl.ac.be/CCI/viewer/download.php
Soils	Soil properties map	FAO Digital Soil Map
Physical infrastructure	Protected area and hydropower site	International Centre for Integrated Mountain Development (ICIMOD) and Department of Hydrological and Metrology (DHM)
Discharge	Daily discharge	Department of Hydrological and Metrology (DHM), Nepal

**Table 2:** Sensitivity ranking of model parameters of SWAT in the Sunkoshi River basin.

Rank	Parameter name	Fitted value	*p* value	*t*-sat
1	CN2	−0.0531	0.001	17.04
2	ALPHA_BNK	0.825	0.001	10.79
3	LAT_TIME	133.740005	0.001	−3.29
4	RCHRG_DP	0.063	0.02	−2.43
5	ALPHA_BF	0.419	1.95	0.05
6	GW_DELAY	13.5	−1.91	0.06
7	SOL_AWC	−0.0009	−1.53	0.13
8	SOL_K	−0.0879	1.47	0.14
9	OV_N	−0.12675	1.27	0.20
10	DEEPST	40650	1.13	0.26
11	SURLAG	2.94795	−1.04	0.30
12	GW_SPYLD	0.01	0.96	0.34
13	SOL_ALB	−0.0537	−0.93	0.35
14	REVAPMN	44.5	−0.85	0.39
15	GW_REVAP	000749	0.82	0.41
16	ESCO	0.005	0.72	0.47
17	SOL_BD	−0.0279	0.70	0.49
18	CH_N2	0.05611	−0.66	0.51
19	SOL_Z	−0.1107	−0.66	0.51
20	_CH_K2	394.565002	−0.54	0.59
21	CANMX	38.1	0.41	0.69
22	SHALLST	30350	0.27	0.79
23	GWQMN	3785	0.25	0.81
24	EPCO	0.455	0.05	0.96
25	SLSUBBSN	14.34	−0.03	0.97

**Table 3:** Monthly calibration and validation statics assessing model performance at one hydrological station.

River	Station (index number)	Index	Calibration	Validation
Sunkoshi	Pachuwarghat (630)	*R* ^2^	0.89	0.79
		NSE	0.88	0.73
		PBAIS	6.31	17.59

**Table 4:** Total power distribution in subbasins.

SN	Subbasin	Catchment area (km^2^)	Power at Q40 (MW)
1	13	322.40	42.29
2	14	175.88	91.87
3	15	685.63	82.77
4	16	628.48	82.83
5	17	197.58	28.90
6	18	119.99	1.58
8	20	108.28	39.84
9	22	138.65	1.20
		Total	371.30

**Table 5:** Hydropower potential at different levels of PoE.

% exceedance	Power (MW)
60	168.00
50	230.58
45	282.47
40	371.30

**Table 6:** Classification of estimated hydropower project Q40 in the Sunkoshi basin.

Hydropower project category based on capacity (MW)	No. of sites	Total power (MW)	% power
≤1	5	2.78	0.74
>1 and ≤10	16	102.04	27.48
>10 and ≤50	15	266.48	71.77
Total	36	371.29	100

## Data Availability

The data will be available from the lead and corresponding author upon special request.
